# Supplementary material for: The NAC Protein from Tamarix hispida, ThNAC7, Confers Salt and Osmotic Stress Tolerance by Increasing Reactive Oxygen Species Scavenging Capability
Source: Plants (Basel). 2019 Jul 12;8(7):221. doi: 10.3390/plants8070221 (PMC6681344; doi:10.3390/plants8070221)
Supplement: Supplementary file 1 [file plants-08-00221-s001.zip › Supplementary Files/Supplementary Tables/Table S1.docx]

**Table S1. Primer sequences used to construct recombinant plasmids**

| **Primer names** | **Primers sequences (5'-3')** |
| --- | --- |
| pROKII-ThNAC7 F | CGCGGATCCATGACTTTACCGGCCCCGAGAC |
| pROKII-ThNAC7 R | CGAGCTCCTACTTAGATGCTTGTGTAGC |
| pFGC5941-ThNAC7-CIS F | CATGCCATGGGAGGAATCATCTTCTGAGTT |
| pFGC5941-ThNAC7-CIS R | TTGGCGCGCCCATCTTGGGTACATCTGCAG |
| pFGC5941-ThNAC7-anti F | CTAGTCTAGAGAGGAATCATCTTCTGAGTT |
| pFGC5941-ThNAC7-anti R | CGCGGATCCCATCTTGGGTACATCTGCAG |
| pBI121-ThNAC7-GFP F | TCTAGACTGGTACCCATGACTTTACCGGCCCCGAGAC |
| pBI121-ThNAC7-GFP R | AGTCAGTCGACCCTTCTTAGATGCTTGTGTAGC |
