# Supplementary material for: The NAC Protein from Tamarix hispida, ThNAC7, Confers Salt and Osmotic Stress Tolerance by Increasing Reactive Oxygen Species Scavenging Capability
Source: Plants (Basel). 2019 Jul 12;8(7):221. doi: 10.3390/plants8070221 (PMC6681344; doi:10.3390/plants8070221)
Supplement: Supplementary file 1 [file plants-08-00221-s001.zip › Supplementary Files/Supplementary Tables/Table S2.docx]

**Table S2. Primer sequences used to construct yeast recombinant plasmids**

| **Primer names** | **Primers sequences (5'-3')** |
| --- | --- |
| pGBKT7-ThNAC7 F | CATGGAGGCCGAATTCATGACTTTACCGGCCCCGAGACG |
| pGBKT7-ThNAC7 R | GCAGGTCGACGGATCCCTACTTAGATGCTTGTGTAGC |
| pGBKT7-ThNAC7_1-435_ F | CATGGAGGCCGAATTCATGACTTTACCGGCCCCGAGACGA |
| pGBKT7-ThNAC7_1-435_ R | GCAGGTCGACGGATCCATGAGAACATGCTCTAGAATCCAC |
| pGBKT7-ThNAC7_146-578_ F | CATGGAGGCCGAATTCAAGAGATGCGTGAATGTCCATAAT |
| pGBKT7-ThNAC7_146-578_ R | GCAGGTCGACGGATCCCTACTTAGATGCTTGTGTAGCAAA |
| pGBKT7-ThNAC7_146-290_ F | CATGGAGGCCGAATTCAAGAGATGCGTGAATGTCCATAAT |
| pGBKT7-ThNAC7_146-290_ R | GCAGGTCGACGGATCCGATGGTAGAGCGTGTCACTGCTCC |
| pGBKT7-ThNAC7_291-435_ F | CATGGAGGCCGAATTCTCTCAAGATCAACATTATAGTGCA |
| pGBKT7-ThNAC7_291-435_ R | GCAGGTCGACGGATCCATGAGAACATGCTCTAGAATCCAC |
| pGBKT7-ThNAC7_436-578_ F | CATGGAGGCCGAATTCGCATATGTTGGAGATATGCTTAGC |
| pGBKT7-ThNAC7_436-578_ R | GCAGGTCGACGGATCCCTACTTAGATGCTTGTGTAGCAAA |
| pGBKT7-ThNAC7_146-215_ F | CATGGAGGCCGAATTCAAGAGATGCGTGAATGTCCATAAT |
| pGBKT7-ThNAC7_146-215_ R | GCAGGTCGACGGATCCCACGTGACTATTGACACCAGAAAT |
| pGBKT7-ThNAC7_216-290_ F | CATGGAGGCCGAATTCCAGGCATCATTGGGTGACCTTGAA |
| pGBKT7-ThNAC7_216-290_ R | GCAGGTCGACGGATCCGATGGTAGAGCGTGTCACTGCTCC |
| pGBKT7-ThNAC7_291-360_ F | CATGGAGGCCGAATTCTCTCAAGATCAACATTATAGTGCA |
| pGBKT7-ThNAC7_291-360_ R | GCAGGTCGACGGATCCTCTGGCCATCTTGGGTACATCTGC |
| pGBKT7-ThNAC7_361-435_ F | CATGGAGGCCGAATTCAAACAAACCCATGTGGCCGATGAT |
| pGBKT7-ThNAC7_361-435_ R | GCAGGTCGACGGATCCATGAGAACATGCTCTAGAATCCAC |
