# Supplementary material for: The NAC Protein from Tamarix hispida, ThNAC7, Confers Salt and Osmotic Stress Tolerance by Increasing Reactive Oxygen Species Scavenging Capability
Source: Plants (Basel). 2019 Jul 12;8(7):221. doi: 10.3390/plants8070221 (PMC6681344; doi:10.3390/plants8070221)
Supplement: Supplementary file 1 [file plants-08-00221-s001.zip › Supplementary Files/Supplementary Tables/Table S3.docx]

**Table S****3. Primer sequences used in real time RT-PCR**

| **Gene** | **GenBank Accession number** | **Forward primers (5'-3')** | **Reverse primers (5'-3')** |
| --- | --- | --- | --- |
| *ThNAC7* | JQ974961 | GAGGAATCATCTTCTGAGTT | AACCATCAGCCATCACAT |
| *Actin* | FJ618517 | AAACAATGGCTGATGCTG | ACAATACCGTGCTCAATAGG |
| *β-tubulin* | FJ618519 | GGAAGCCATAGAAAGACC | CAACAAATGTGGGATGCT |
| *ThPOD1* | KF756934 | GTGCTGATATCGTGATCATG | ATTGGTTGTAGAGGCGGTAG |
| *ThPOD2* | KF756935 | TGACCTCGTTGCACTATCAG | GAATAAGCCTTCGTCATCAG |
| *ThPOD3* | KF756936 | ACGTTGAGCTAGGGAGAAG | GAGGTTATCATCGCTTCCT |
| *ThSOD1* | KF756930 | TCATCAACATCTAATGTTCC | CGTAAAGCTAGGTTGATTGG |
| *ThSOD2* | KF756931 | TCTTCGAGCAGCTCCAGCTT | CTCGTCCTCATACTATCCAT |
| *ThSOD3* | KF756932 | GTACACAGTGGTATTTCCAG | CTCTGAATACAATCAGTGTC |
| *ThP5CS1* | KM101096 | GAGTACAGTTCACTTGCTTG | GAGAAGCCATCTTGTGGTT |
| *ThP5CS2* | KM101097 | CAGAATGGCCTTATGGCTCT | GACCTTCTACATCACTCAGC |
| *actin 3* | AT3G53750 | CATGCAATCCTACGTCTTG | TCAGATCCAATGGTGATCAC |
| *α-tubulin* | AT1G50010 | GATGTACCGTGGTGATGTC | GAGCCTCTGAAAATTCTCC |
| *POD* | AT1G14550 | CCATAGGACAATCTCAATGC | TGATCGGTTACTAATAG |
| *POD* | AT2G18140 | TCCGGGAGCCACACCATTGG | TGGTCGGAATTCAACAG |
| *POD* | AT5G58400 | GGCAAGCCAGGTGCGTCAC | CCGGCTGTAGGATACGAC |
| *SOD* | AT1G08830 | ATGTCTACTGGTCCACATTTC | ATGGCCTCCCTTTCCGAG |
| *SOD* | AT2G28190 | ATGTATCTCAACAGGACCAC | AGTGGTCAGACTAAGCTC |
| *SOD* | AT3G10920 | AGTGAAGGTGGTGGAGAGC | CATCTATACCCACCAGAG |
| *P5CS1* | AT2G39800 | ACCAGAAGCACGGTCATTC | CCATCTGAGAATCTTGTG |
| *P5CS2* | AT3G55610 | ATGATCTTATTTATGTTCTGC | CACTATCTTCCGTCACTAT |
