# Supplementary material for: The NAC Protein from Tamarix hispida, ThNAC7, Confers Salt and Osmotic Stress Tolerance by Increasing Reactive Oxygen Species Scavenging Capability
Source: Plants (Basel). 2019 Jul 12;8(7):221. doi: 10.3390/plants8070221 (PMC6681344; doi:10.3390/plants8070221)
Supplement: Supplementary file 1 [file plants-08-00221-s001.zip › Supplementary Files/Supplementary Tables/Table S6.docx]

**Table S6. The primer sequences of qRT-PCR for validation of the selected DEGs**

| **GenBank Accession number** | **Forward primers (5'-3')** | **Reverse primers (5'-3')** |
| --- | --- | --- |
| AT3G53750 | CATGCAATCCTACGTCTTG | TCAGATCCAATGGTGATCAC |
| AT1G50010 | GATGTACCGTGGTGATGTC | GAGCCTCTGAAAATTCTCC |
| AT5G42180 | AACTACCAGCTCCTACTTTC | CATTCTTCACCGTGTTATGG |
| AT5G24240 | TGACTGGACCACGAACTT | GTCTAGTACTGATATCTTGTG |
| AT5G57550 | TACCATGCATACGAATGTGT | CGTTCCATAGACTCGAGTAT |
| AT1G75750 | TGTTCTCCAACTCGTCCAGG | CACTGGCACTTGTCGTAGTT |
| AT2G38240 | ATGGTGAACCATGGTGTG | CTTATGGAAGAAGGCAAGT |
| AT1G43160 | GGTTCAGCTGTGACTAAAG | TGCTTTGCTTCCTCTAAAGC |
| AT4G22485 | CAGAAAGTCCTCCAAACACT | AGGTGTTTCTGGTGGAGT |
| AT2G34390 | CCACCTCTCTGCACATTTC | GATGAACTCCATCACGAATG |
| AT1G50750 | TTGCTGAGAAATGGTGTCCT | ATCTCTCCATCCATGCTACT |
| AT2G26010 | ATGGCTAAGTCTGCTGCCAT | TTAACATGGGAAGTAACAG |
| AT4G29610 | AACCTCGCTCTCAACTCCAT | AGAAGGAGGGAGGCATCTT |
| AT5G52750 | ATGACAGTAGTTGGTGAAGT | CATAGGCAGGATTGTATTGG |
