# Supplementary figures and images for: The NAC Protein from Tamarix hispida, ThNAC7, Confers Salt and Osmotic Stress Tolerance by Increasing Reactive Oxygen Species Scavenging Capability
Source: Plants (Basel). 2019 Jul 12;8(7):221. doi: 10.3390/plants8070221 (PMC6681344; doi:10.3390/plants8070221)

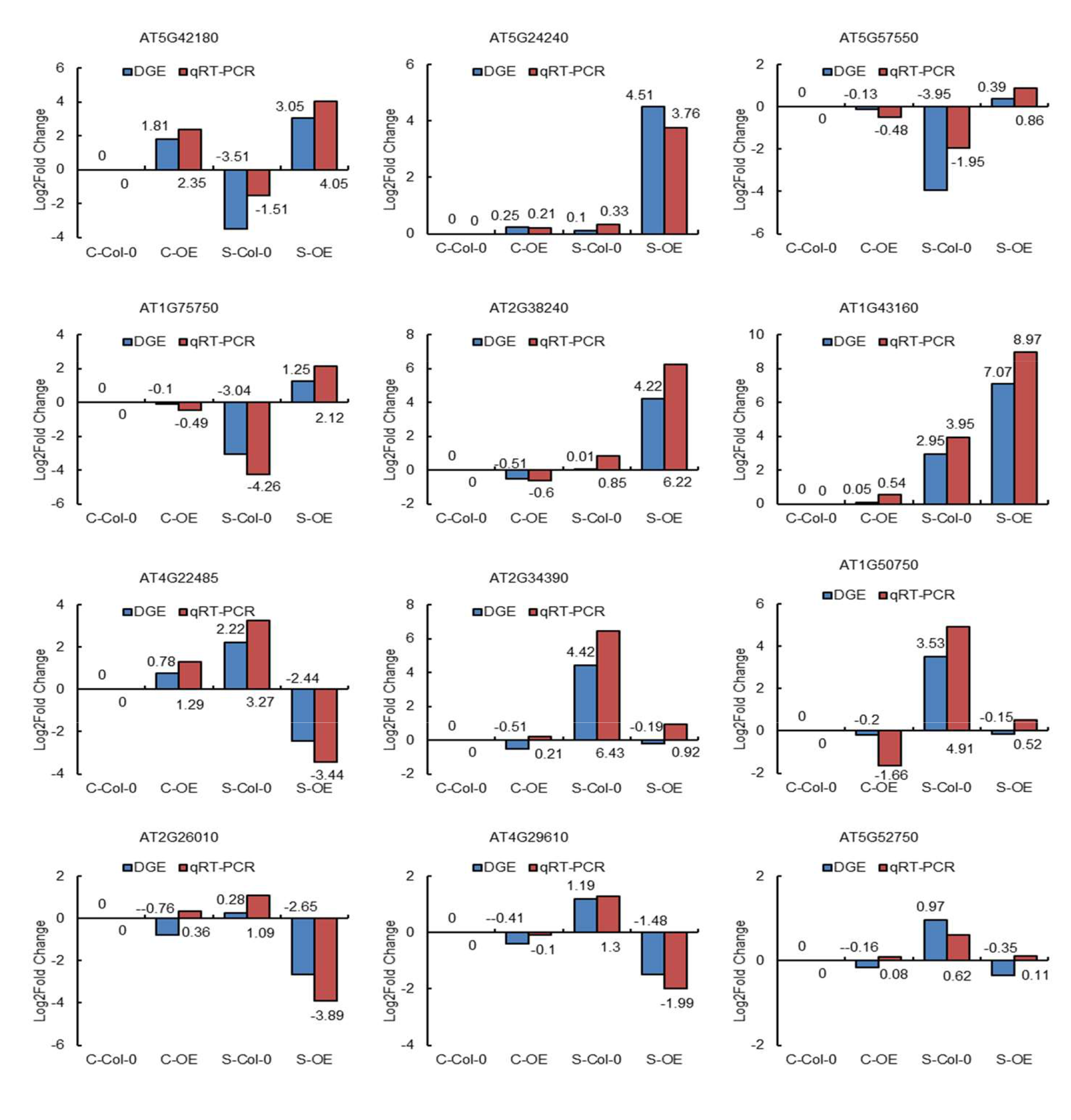

Supplement: Supplementary file 1 [file plants-08-00221-s001.zip › Supplementary Files/Supplementary Figures/Figure S10.tif]

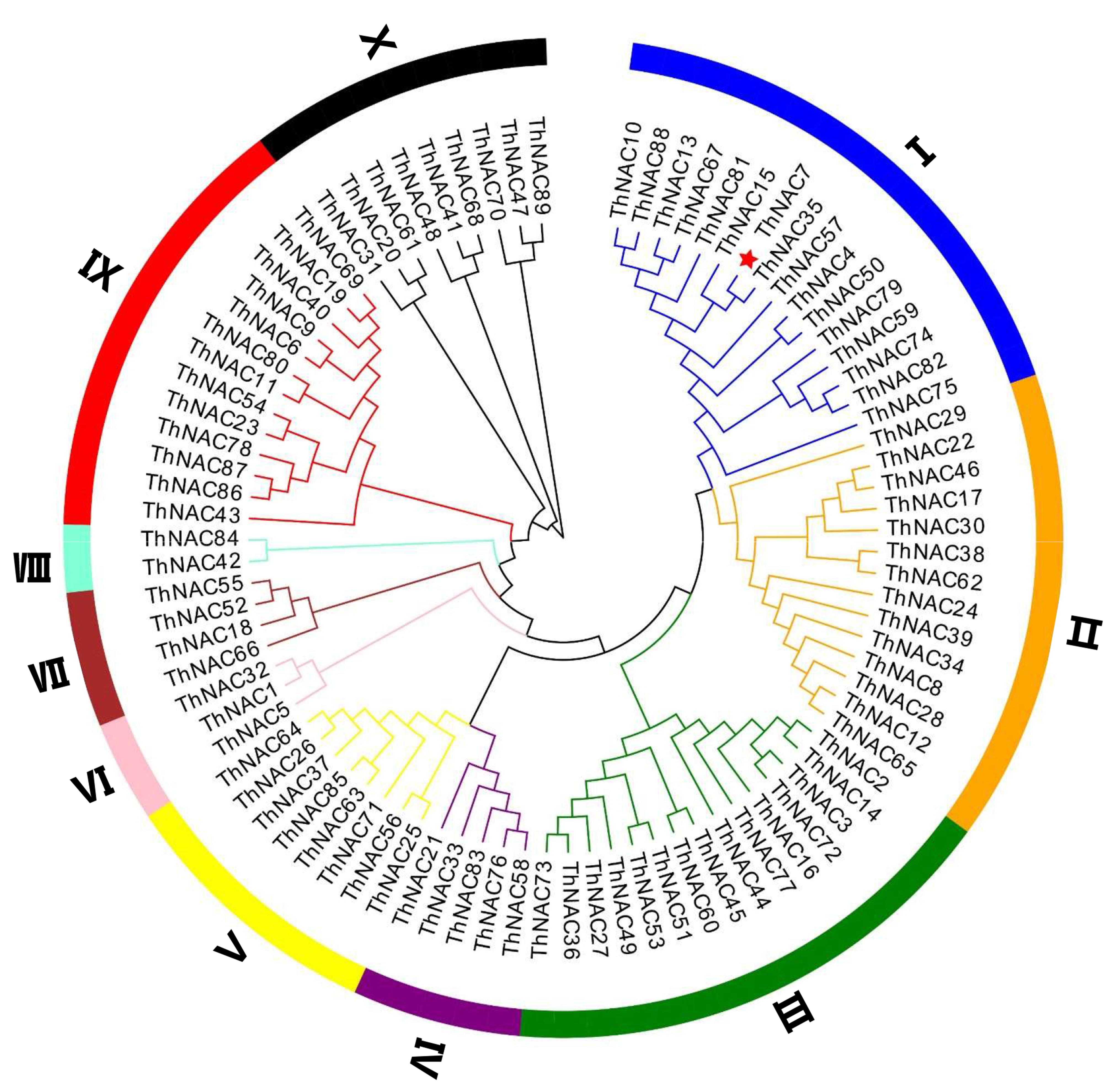

Supplement: Supplementary file 1 [file plants-08-00221-s001.zip › Supplementary Files/Supplementary Figures/Figure S2.tif]

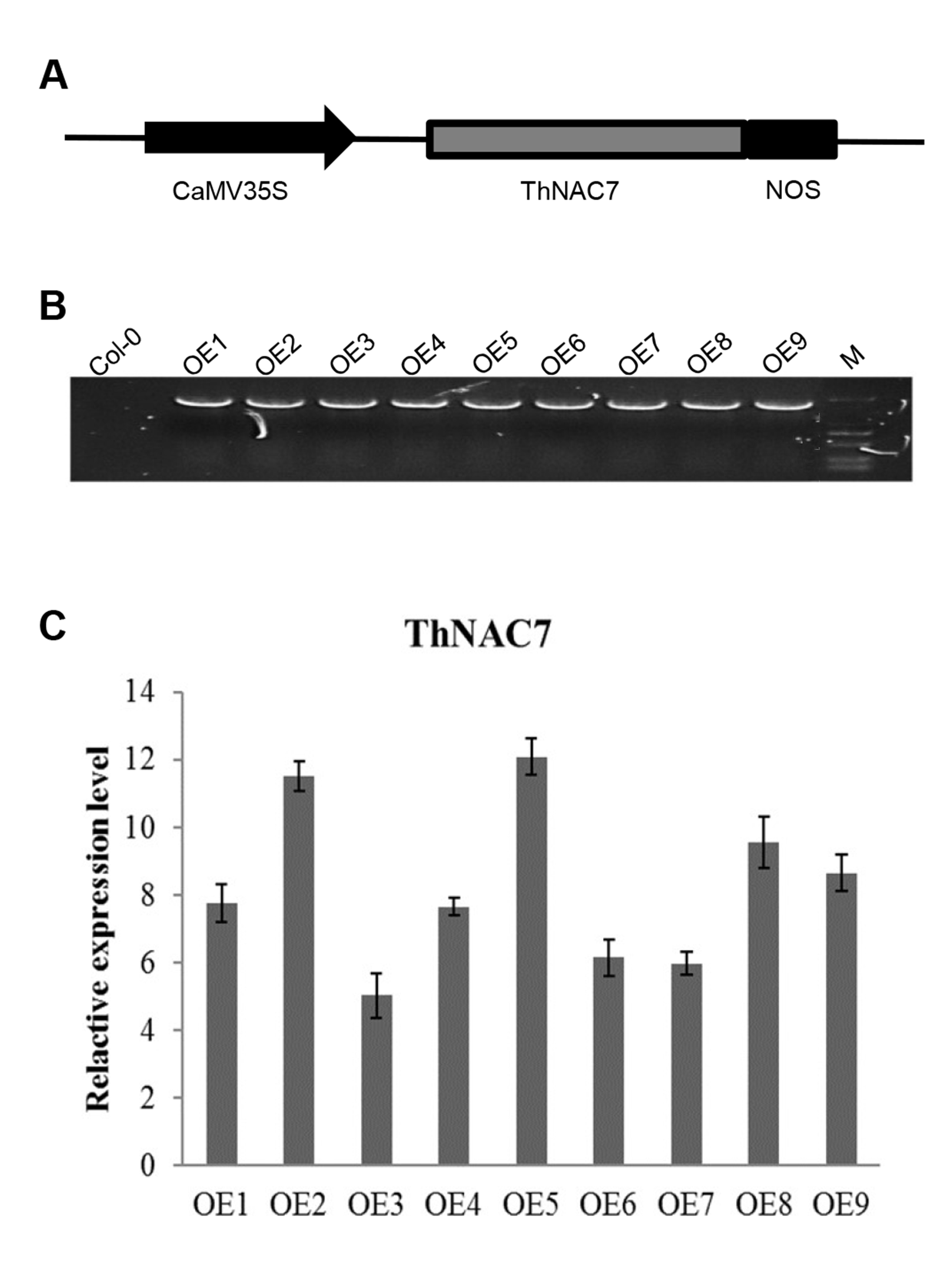

Supplement: Supplementary file 1 [file plants-08-00221-s001.zip › Supplementary Files/Supplementary Figures/Figure S3.tif]

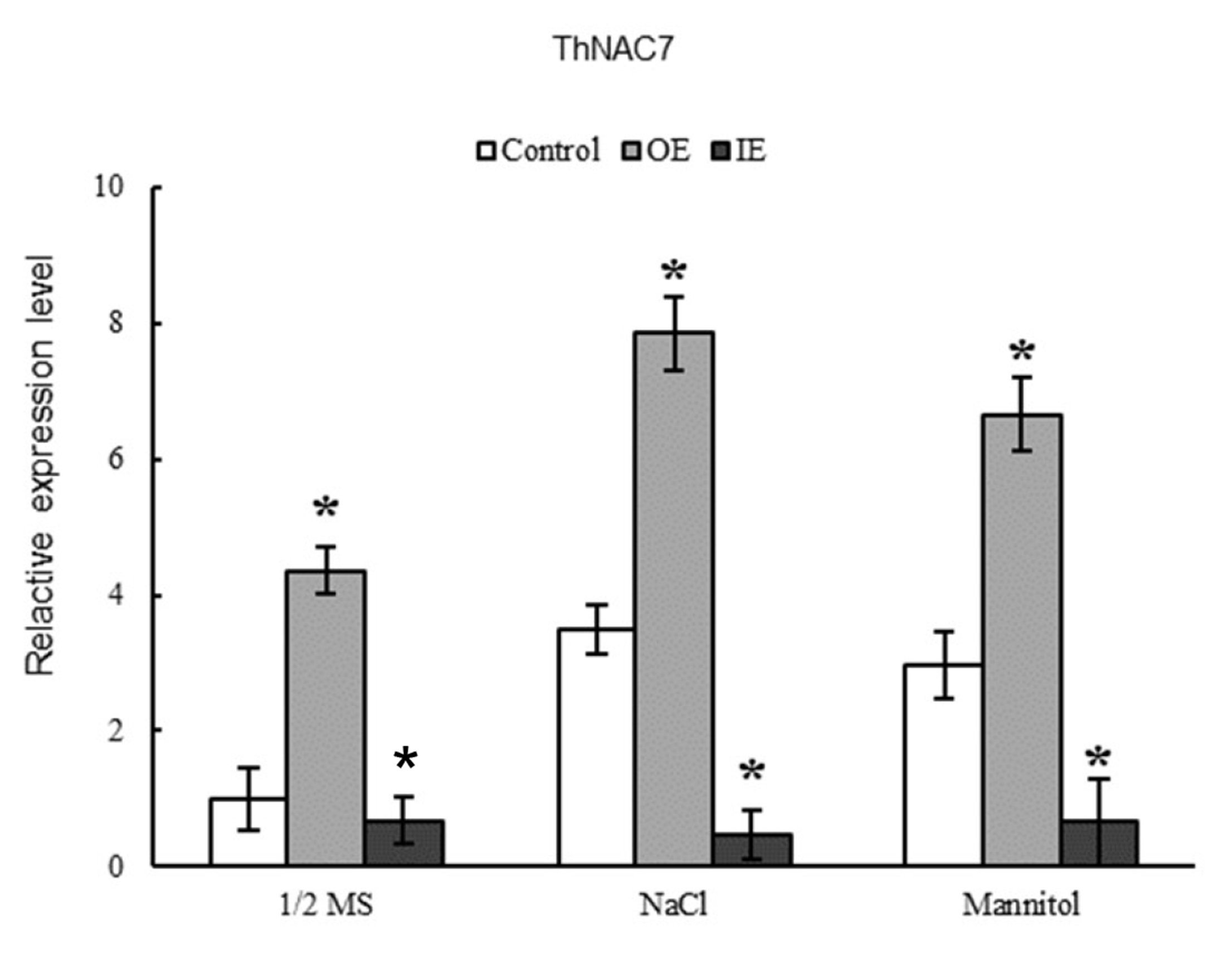

Supplement: Supplementary file 1 [file plants-08-00221-s001.zip › Supplementary Files/Supplementary Figures/Figure S4.tif]

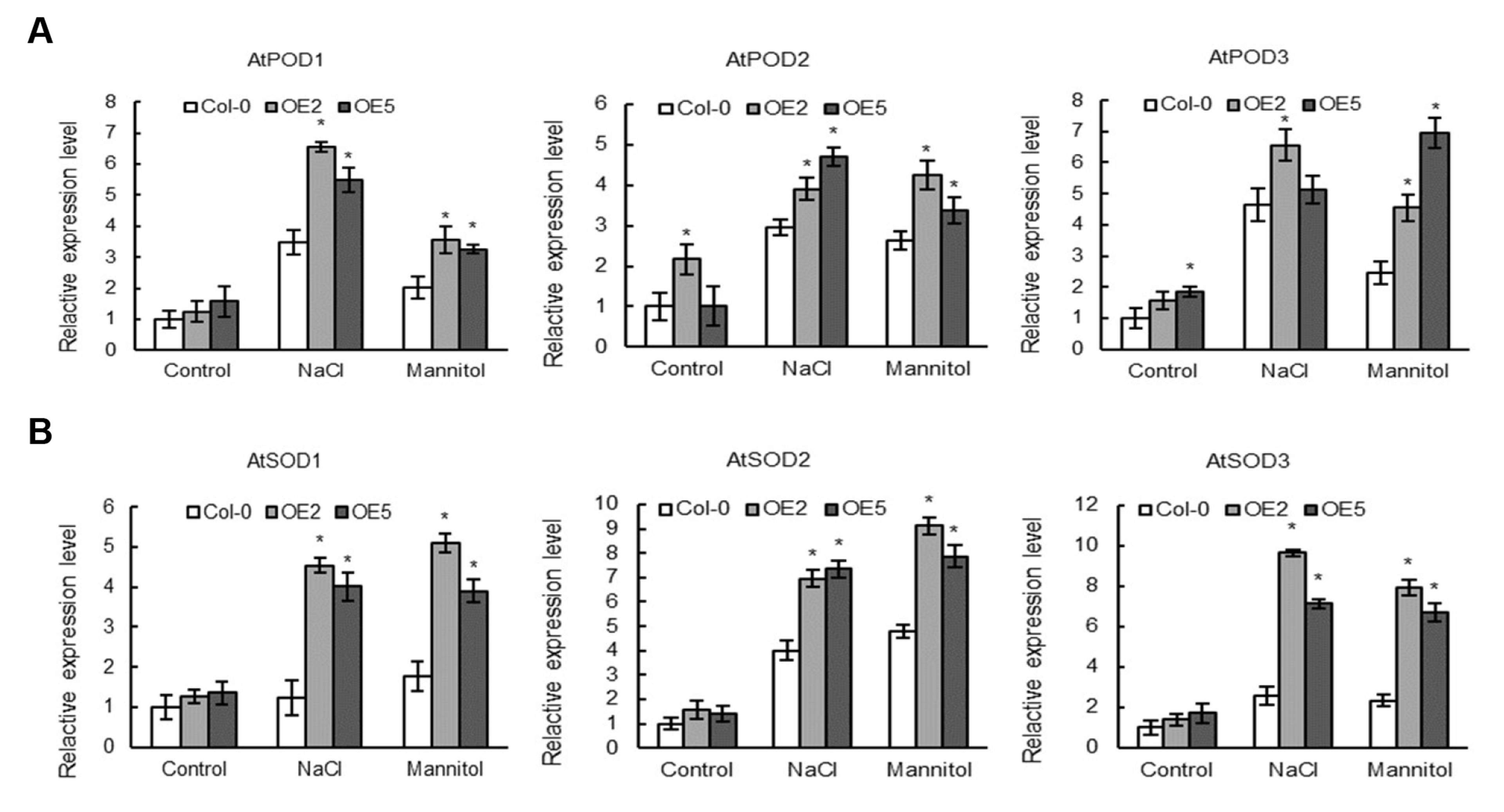

Supplement: Supplementary file 1 [file plants-08-00221-s001.zip › Supplementary Files/Supplementary Figures/Figure S6.tif]

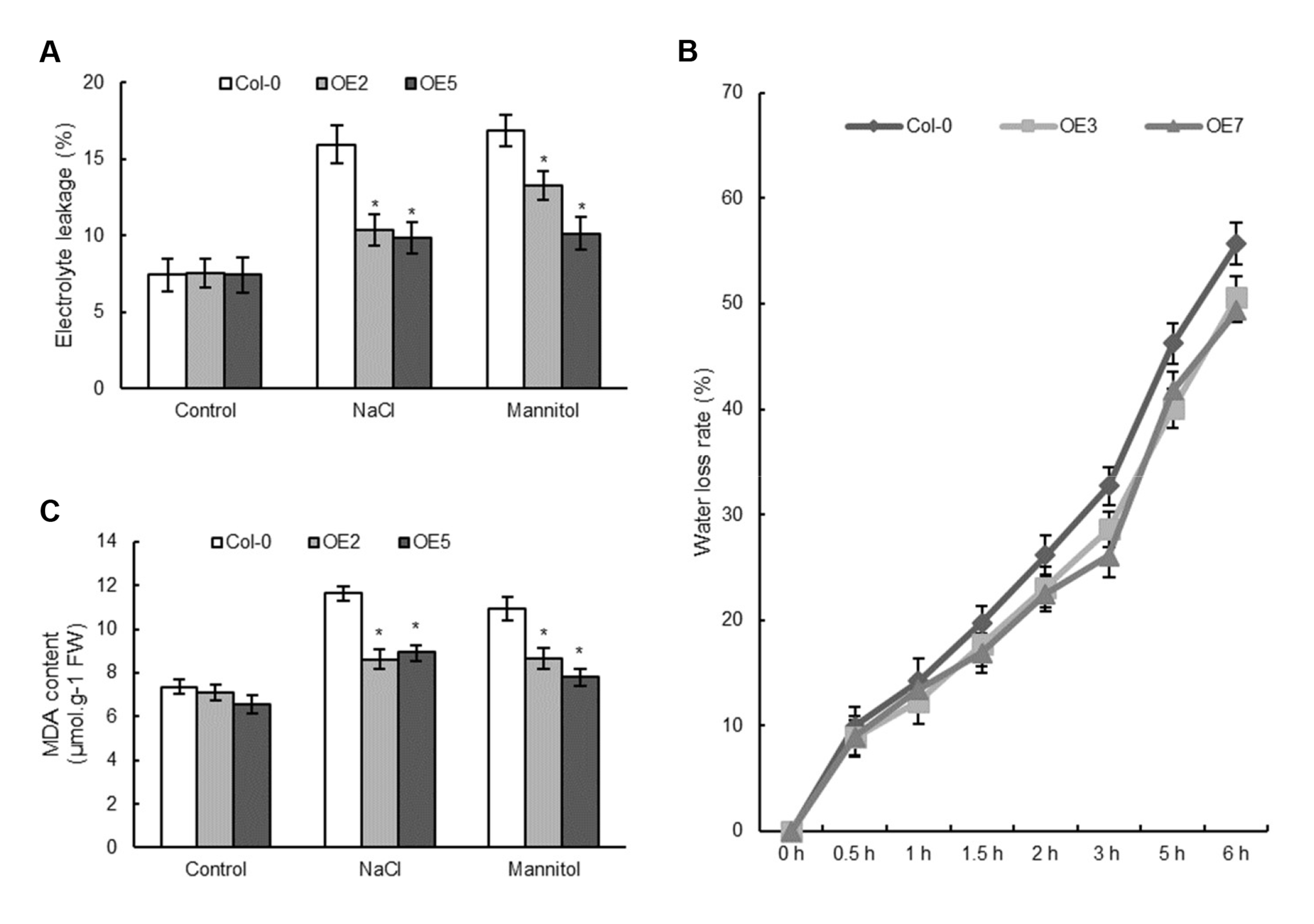

Supplement: Supplementary file 1 [file plants-08-00221-s001.zip › Supplementary Files/Supplementary Figures/Figure S7.tif]

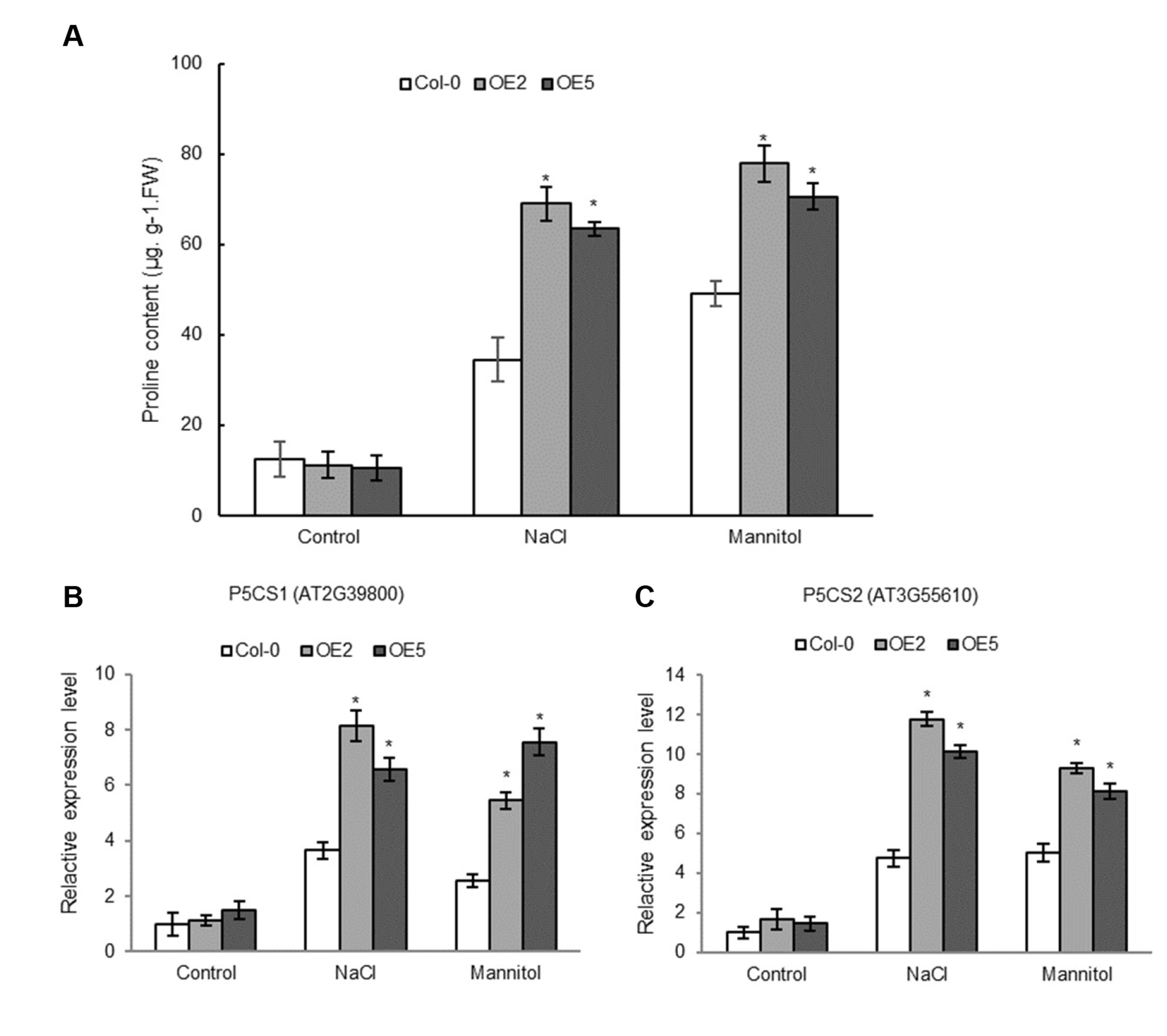

Supplement: Supplementary file 1 [file plants-08-00221-s001.zip › Supplementary Files/Supplementary Figures/Figure S8.tif]

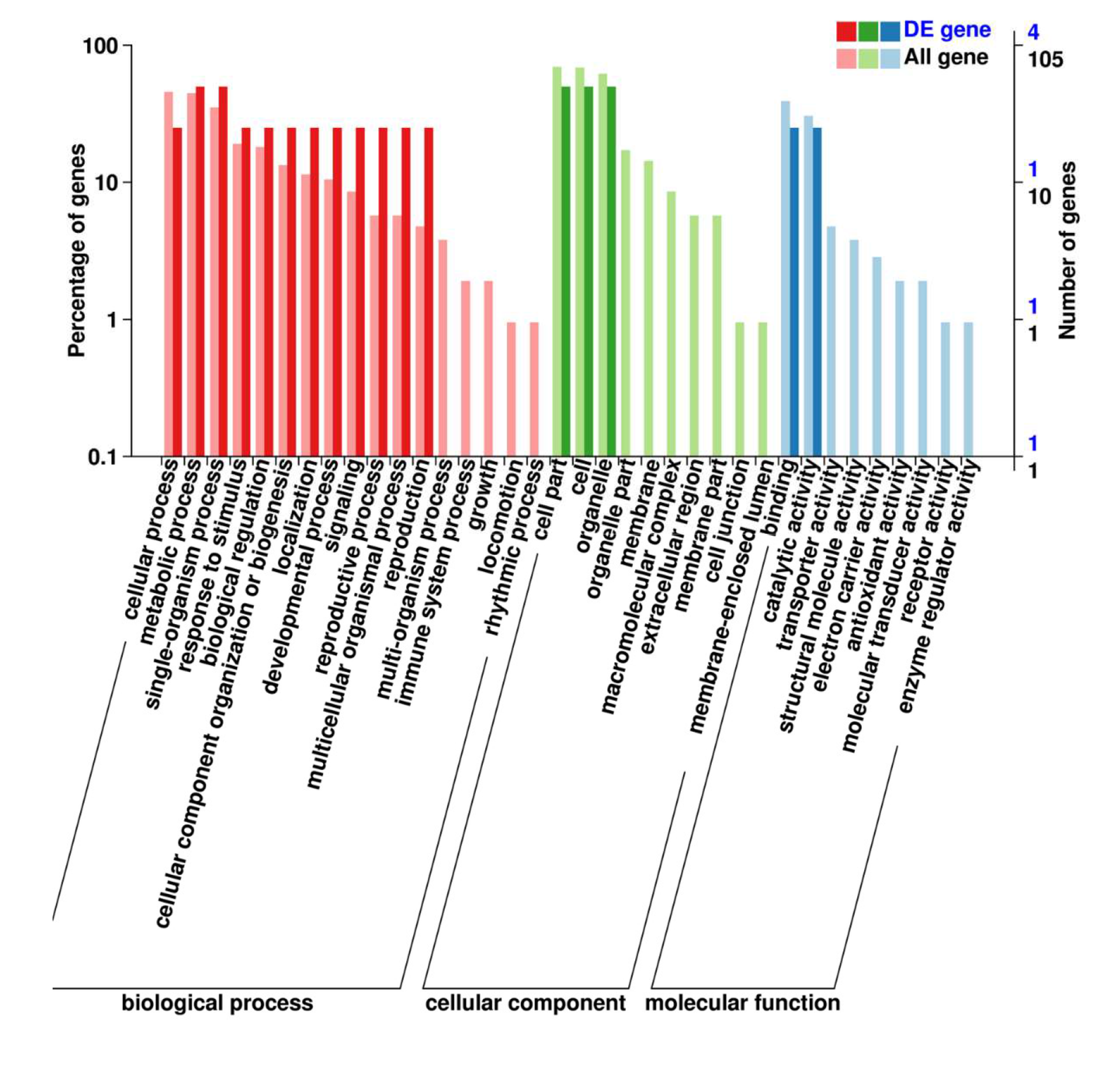

Supplement: Supplementary file 1 [file plants-08-00221-s001.zip › Supplementary Files/Supplementary Figures/Figure S9.tif]
